# Supplementary material for: Physalia physalis—A Source of Bioactive Collagen for the Cosmetic Industry
Source: Int J Mol Sci. 2025 Dec 19;27(1):33. doi: 10.3390/ijms27010033 (PMC12785602; doi:10.3390/ijms27010033)
Supplement: Supplementary file 1 [file ijms-27-00033-s001.zip › ijms-4001566-supplementary.pdf]

Supplementary Figures

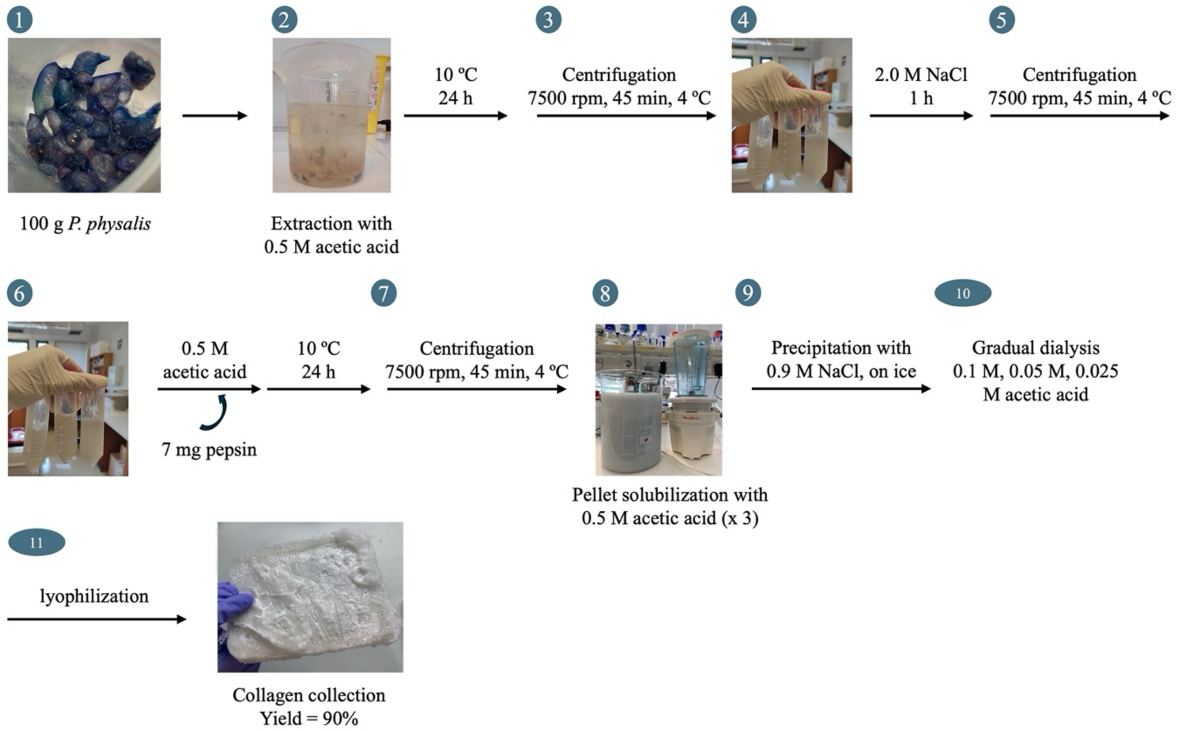

Supplementary Figure S1: Schematic representation of collagen extraction from *Physalia physalis*.

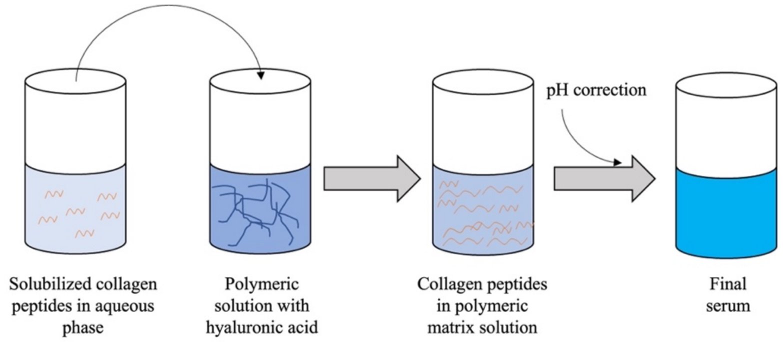

Supplementary Figure S2: Serum development with incorporation of 0.50% collagen biopeptides using a polymeric solution containing hyaluronic acid. The figure illustrates the formulation workflow, including dissolution of collagen hydrolysates in the aqueous phase, incorporation into the polymeric matrix, pH adjustment, and viscosity optimization. During a 30-day accelerated stability test (4–45 °C cycling), no phase separation was observed, and the serum maintained a homogeneous appearance and consistent rheological properties, indicating the formation of an adequate and stable polymeric network.

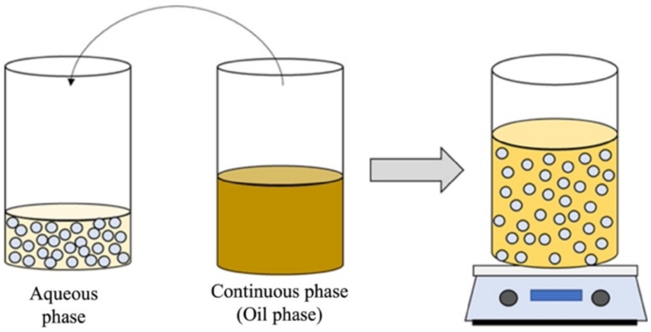

Supplementary Figure S3: Water-in-oil nanoemulsion-based serum product containing 0.15% of collagen biopeptides. Visual stability assessments confirmed that the nanoemulsion maintained its structural integrity (at 4–45 °C cycling) and exhibited no phase separation throughout the testing period, reflecting the effectiveness of the selected surfactant system and its optimized ratios.
